# Predicting green: really radical (plant) predictive processing

**DOI:** 10.1098/rsif.2017.0096

**Published:** 2017-06-21

**Authors:** Paco Calvo, Karl Friston

**Affiliations:** 1 EIDYN Research Centre, and Institute of Molecular Plant Sciences, University of Edinburgh, Edinburgh, UK; 2 MINT Lab, Departamento de Filosofía, Universidad de Murcia, Murcia, Spain; 3 Wellcome Trust Centre for Neuroimaging, Institute of Neurology UCL, 12 Queen Square, London, UK

**Keywords:** plant intelligence, free energy, predictive processing, perceptual/active inference, embodiment, affordance

## Abstract

In this article we account for the way plants respond to salient features of their environment under the free-energy principle for biological systems. Biological self-organization amounts to the minimization of surprise over time. We posit that any self-organizing system must embody a generative model whose predictions ensure that (expected) free energy is minimized through action. Plants respond in a fast, and yet coordinated manner, to environmental contingencies. They pro-actively sample their local environment to elicit information with an adaptive value. Our main thesis is that plant behaviour takes place by way of a process (active inference) that predicts the environmental sources of sensory stimulation. This principle, we argue, endows plants with a form of perception that underwrites purposeful, anticipatory behaviour. The aim of the article is to assess the prospects of a radical predictive processing story that would follow naturally from the free-energy principle for biological systems; an approach that may ultimately bear upon our understanding of life and cognition more broadly.

## 1. Introduction

The emergent field of ‘plant neurobiology’ [1,2] studies the flexible and adaptive behaviour of plants beyond the domains of plant biochemistry/cellular and molecular biology mechanistic approaches. The cognitive sciences have, up until now, been neglectful of the plant world. But, as it turns out, plants exhibit marks of intelligence [3], and are thus subject, in principle, to theoretical and empirical scrutiny with tools that have been hitherto restricted to the cognitive sciences [4].

The behaviour of plants is reversible, and soft-wired in a manner that responds to metabolically salient features of the environment [5]. Plants can navigate many vectors (not just light and gravity), where the implicit multimodal integration partly accounts for the adaptive responses observed. The capacity for foresight may be crucial to optimize fitness: some roots and shoots can anticipate the future, competing for patchily distributed resources, and growing, branching and flowering differentially depending upon the prospective acquisition of minerals, water and light, among many other vectors [6]. For instance, many plants can anticipate future shade, initiating phenotypic changes in response to far-red/red light cues ahead of time [7]. *Cuscuta* (dodder) attaches to plants, coiling around them and developing haustoria that can penetrate their hosts' vascular systems and thereby suck up nutrients. *Cuscuta* optimizes fitness by deciding how much energy to invest in coiling around a host with respect to the overall nutrient intake yet to be obtained [8]. Many more examples of decision-making—that anticipates reward—exist in the literature [7–11], but for present purposes we only need to bear in mind that plants (appear to) exhibit anticipatory, goal-directed behaviour.

In this context, the philosophy of plant neurobiology [4] proposes to assess plant intelligence by framing an integrated view of plant signalling and adaptive behaviour, and to account for the way plants perceive and act *purposefully* under different paradigms. It is in this setting that we consider the theoretical possibility that plants, like animals, are ‘predictive processors’ [12]. Although with an eye to the design of experimental protocols, such prospects have already been explored elsewhere [13],^1^ in a further twist, we elaborate on the possibility that plant neurobiology may throw a distinctive light upon the more or less radical form that such predictive machinery may take. The overall aim of this paper, once zoo-centric and neuro-centric biases have been set aside, is to assess the prospects of a *really radical (plant) predictive processing*; an approach that may ultimately bear upon our understanding of life and cognition more broadly.

## 2. The predictive processing basics, thus far

The notion of a general (biotic) radical predictive processing story follows naturally from the free-energy principle for biological systems [14]. The free-energy principle is the mathematical back-story behind predictive processing and active inference in the neurosciences [15]. In brief, the free-energy principle starts with the premise that biological systems resist the second law of thermodynamics by restricting themselves to a small number of attracting states. In other words, the nature of living things can be expressed in terms of a high probability of being in a small number of (characteristic) states and a very low probability of occupying all other states. This somewhat abstract notion has some important if subtle implications. For example, if we define *surprise* (also known as *self information* or *surprisal*) as the negative log probability that a plant—or any biological system—will be found in a particular state, then one can cast biological self-organization as minimizing surprise over time. Formally, the time average of *surprise* or *surprisal* is called an *action*. This means that biological systems conform to Hamilton's principle of least (or more exactly, stationary) action.

The (time) average of surprise is also known as *uncertainty* (or *entropy* in information theoretic terms). This means that the defining hallmark of biological systems is a tendency to resolve uncertainty or, from the point of view of stochastic thermodynamics, minimize entropy (hence resisting the second law). Free energy gets into the game by providing a proxy or bound approximation for surprise that living systems can minimize explicitly [16]. Under certain simplifying assumptions, the prediction error—that is celebrated in predictive coding formulations of predictive processing [17,18]—can be regarded as the free energy or surprise. There is one final interpretation of surprise that we will appeal to later. Mathematically, surprise is the negative log marginal likelihood or Bayesian model evidence. This means that by minimizing surprise, free energy or prediction error, a plant—and indeed you—maximizes Bayesian model evidence. In other words, plants behave like little statisticians, making implicit inferences about their world through changes in their internal states. In cognitive neuroscience, this leads to the Bayesian brain hypothesis [19–23]. So what does this (Bayesian plant hypothesis) mean for self-organization in plants?

From a technical perspective, there is no distinction between a brain, a plant, a cell or organelle [23,24]. All are just examples of systems that self-organize according to the free-energy principle. In other words, they exchange with their environment in a way that minimizes surprise and resolves uncertainty. Generally, this reduction of surprise can be described from two perspectives; reflecting the circular causality induced by an embodied exchange of the organism with its environment. These two processes correspond to *perceptual* and *active* inference. Perceptual inference corresponds to adjusting internal states to provide better predictions, while active inference is the complementary process of sampling the environment to make (sensory) samples match predictions. Perceptual and active inference entail each other—both in the service of minimizing surprise. From the outset, one can see that active inference has an enactive or embodied aspect that is a necessary consequence of any sentient system in (thermodynamically open) exchange with its environment [14].

Two key issues will figure centrally in our arguments. The first pertains to the notion of a *generative model*. Irrespective of the particular processes in play, the notion of a prediction calls upon a model that generates predictions. This is usually associated with the internal states of a creature or plant that come to recapitulate causal structure in external or environmental states generating sensory samples. This recapitulation does not necessarily have to be isomorphic and may, or may not, be representational in nature. However, in accord with the good regulator theorem [25], the one thing we know is that the embodied structure of any biological system must in some way be a good match for the eco-niche in which it is immersed.

**Figure 1.**
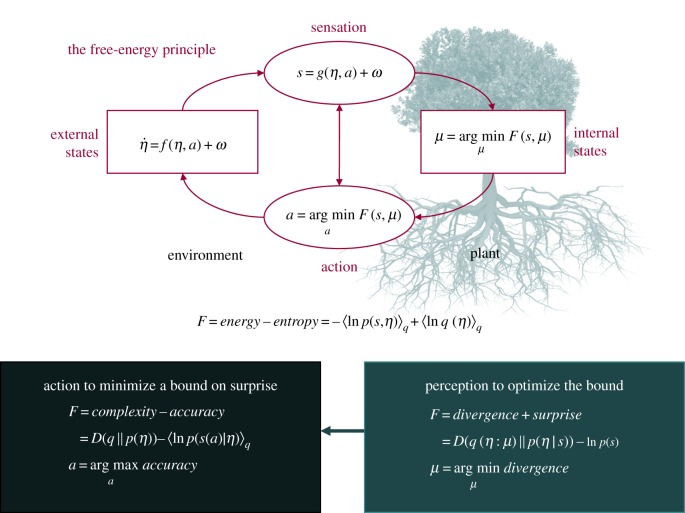
Upper panel: schematic of the quantities that define free energy. These include the internal states of a system *μ* (e.g. a plant) and quantities describing exchange with the world; namely, sensory input 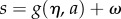 and action *a* that changes the way the environment is sampled. The environment is described by equations of motion, 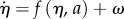, that specify the dynamics of (hidden) states of the world *η*. Here, *ω* denotes random fluctuations. Internal states and action both change to minimize free energy, which is a function of sensory input and a probabilistic representation (recognition density) 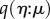 encoded by internal states. Lower panel: alternative expressions for the free energy illustrating what its minimization entails. For action, free energy can only be suppressed by increasing the accuracy of sensory data (i.e. selectively sampling data that are predicted by the representation). Conversely, optimizing internal states make the representation an approximate conditional density on the causes of sensory input (by minimizing divergence). This optimization makes the free-energy bound on surprise tighter and enables action to avoid surprising sensations. (Online version in colour.)

The second issue is the nature of free energy or prediction error minimization. This can be cast at a number of levels that speak to an inherent anticipatory exchange with the environment. At its simplest, the minimization of surprise can be regarded (in dynamical systems) as a gradient descent. Crucially, this gradient descent progresses in generalized coordinates of motion (for example, in terms of position *and momentum*) [26,27]. This lends the suppression of prediction errors a dynamical and anticipatory aspect; in the sense that a sentient system is not simply reducing prediction error but pursuing trajectories that have the least free energy. In other words, it is not just a question of changing to reduce surprise but choosing actions that will reduce *expected surprise* in the future (notice that expected surprise is, mathematically, the same as the average surprise which, we have established, is entropy or uncertainty). We will see a nice example of this below when we consider how plants respond to time-dependent changes in salt concentrations. One can take this argument to its extreme and posit that any self-organizing system must embody a generative model whose predictions ensure that *expected free energy or uncertainty is minimized through action* [28]. Our basic argument is that this principle endows plants with a purposeful behaviour that is quintessentially anticipatory in nature. See figure 1 for a summary of the technical principles that underlie active inference and free-energy minimization.

## 3. Plant predictive processing—or ways to avoid salty surprises

Because the overt behaviour of plants is in general not obvious to the naked eye, we tend to think of them as bottom-up energy receptacles; information-processors that just sit back and wait for the world to bring news hopefully relevant to their doings. But to survive, plants rely on the veridical perception of their surroundings. To do so, predicting their own states of sensory stimulation, and not merely reacting to them, proves critical [12]. In this way, plants are subject to scrutiny under the light of the general predictive processing principles just reviewed. Since active inference suggests some form of agency, plants, like animals, appear to be engaged in pro-active sampling of their local environment for the sake of generating information with an adaptive value (i.e. reducing uncertainty or avoiding surprises). In fact, plants are constantly, and tirelessly, swaying their organs towards energy gradients, doing their best to realize the most likely (least surprising) implications of the surrounding sensory stimulation for subsequent engagement with the world.

The main thesis of plant predictive processing (PPP) is that plant behaviour takes place by way of a process that predicts the environmental sources of sensory stimulation. According to this picture, a plant's predictions would propagate backwards to its effector organs (substrate details are reviewed in the next section). When a mismatch between the prediction generated and the incoming signal takes place, that is, when there is a prediction error, the mismatch is propagated upwards, into the plant internal infrastructure. With this strategy, plants could, in principle, minimize the divergence between the predictions (empirical bets) about sensory signals and the actual signal supplied by the environment. Plant perception could thus boil down to a process whereby environmental input is matched to top-down predictions. There are several (free energy compliant) schemes one could invoke to explain this behaviour. Perhaps the simplest and subtlest is to simply ‘explore’ when prediction errors are high. In other words, when predictions match signals from the plants, actively sampling of the eco-niche is suspended (no more error needs to be minimized). Conversely, if prediction errors are high, the plant ‘knows’ its current ‘behaviour’ is producing surprising outcomes and that there is at least one surprise reducing, prediction fulfilling response; namely, ‘get out of here’. This simple form of optimization is ubiquitous in self-organization: in theoretical evolution, it is formally related to second order selection (also known as selection for selectability) [29,30]. In cognitive neuroscience, it has been referred to in terms of autovitiation (i.e. the destruction of self-induced but surprising fixed points of attraction) [31]. Genetic algorithms that use this sort of scheme include adaptive stochastic optimization procedures. Remarkably, recent advances in these optimization schemes have been inspired by plant (weed) behaviour [32]!

Note, we are not simply saying that plants indulge themselves using adaptive stochastic optimization, we are saying that there are simple but powerful schemes that can resolve the prediction errors afforded by the capacity to predict. This or related forms of self-organization based upon predictions are what we end up calling active ‘plant perception’. It basically boils down to the selection of actions that best minimizes surprise expected under those actions. This means that behaviour is driven by the resolution of uncertainty—with an eye to ensuring that a flexible, surprise avoiding, sampling of sensory inputs fits their plastic phenotype (or, in free-energy parlance, fits prior beliefs embodied in their physical structure). In this way, plant perception entails predictive hypotheses as to what is out there: it could be a herbivore representing bad news [33], it could be a stream of water [34], or what may.

Anticipatory plant behaviour has been tested at the root level with, for example, pea roots responding in a timely way to environmental contingencies. In particular, pea plants exposed to dynamic nutrient regimes are observed to develop more roots in patches with an *increasing* level of nutrients, despite those patches being less rich in absolute terms than others that were nevertheless static (with no nutrient increase). As [7] observes: ‘These findings demonstrate that rather than responding to mere absolute resource availabilities, plants are able to perceive and integrate information regarding dynamic changes in resource levels and utilize it to anticipate growth conditions in ways that maximize their long-term performance’ (p. 63). Roots don't simply just grow. They constantly assess the (future) acquisition of minerals and water. Or consider, for the sake of illustration under PPP, halotropic (salt-avoiding) root behaviour [35]. Ionic/osmotic stress brought about by high concentrations of salt in the soil can disrupt, rather badly, the biochemical machinery of plant cells, affecting photosynthesis rates and protein synthesis, among other key processes. Roots have also been observed to exhibit random agravitropic responses under salt stress [35]. Generally speaking, salt means high ‘surprise’ to plants. In this way, we may cash out roots' exposure to salt in terms of surprise states. How then are we to proceed?

First, we need to bear in mind that being in a state of surprise, or not, is specific to each phenotype and individual. We tend to think of plants as a monolithic category, but different plants (not to mention different plant species) have their own needs and their own means to get around. Put simply, what is surprising for one plant species/individual may not be for another. In fact, salt-avoidance may even mean a comforting familiar (low surprise) outcome to some species! Think of land species based in seashore intertidal zones, estuaries, and the like, which have adapted to tolerate salt.^2^

Plants have a number of tools at their disposal to cope with salt stress. Some plants efflux salt from shoot meristems and from the more active leaves, in terms of photosynthetic potential. Those unable to do so have developed tolerance strategies that rely on the maintenance of ion homeostasis and the retention of water, among other mechanisms [37]. They can also abscise leaves under osmotic stress. Mangroves make osmotic adjustments accordingly. Or take saltbushes (*Atriplex* sp.). They have glands that retain salt in the leaves, becoming harmless as it crystallizes [38]. Surprise and entropy, one can see, can vary wildly. In short, plants will try to avoid states that are surprising—but the specification of what is surprising ‘makes the plant’. In our salt-avoidance example, the (implicit) belief that salty outcomes are rare is constituted by the (explicit) phenotype of the plant.

According to PPP, cascades of sensory input may be surprising to plants, generating a prediction error. A surprising state for a plant can be one in which it is too salty, or too dry, to survive. The working hypothesis of PPP therefore is that plants have an architecture conceived as an *anticipatory engine*. If plants are pro-active, anticipatory engines they are constantly looking ahead, monitoring gradients, *guessing* ahead of time what the world is like—so as to adapt to local conditions via phenotypic plasticity. The aim of the plant is then to minimize discrepancies, to reduce error as much as possible, and this can be accomplished through perceptual or active inference. Under *perceptual inference*, prediction errors can be minimized by updating predictions so that they are brought into line with actual sensory states. As prediction error is minimized, the plant root system may be said to have perceived the (gradient of) salt concentrations of the cause of stress. Once the plant updates its expectations, it expects to be in salt-water, and if the levels are tolerable, adjust by triggering morphological changes (e.g. hardening responses to abiotic stresses) in response to sensory states.

But ‘inferring the world is a hard place’, so to speak, is not the only solution available. Probably, if you are the type of plant that finds itself on the seashore, perceptual inference is not the best adaptive strategy. Roots may keep track of the salt gradient, and turn from the source of salt stress. In this way, plant roots can resample via *active inference*. Rather than updating expectations that underwrite predictions, the plant may choose to sample more selectively the sensory states through nutation movements of the roots—so that they match the expectations that the plant *had*. Plants can thus re-sample their vicinity with a halotropic response that brings things back on track, into line with predictions and prior expectations. If the reader finds plant examples along these lines a hard pill to swallow, just think of animal vision, where saccades bring about the sampling of sensory states. In the same way that evidence can be gathered by visual saccading to make predictions about visual input [39], a full-fledged active-inferential theory of root nutation states that nutations constitute the sampling of sensory states, and that by taking in different parts of the soil structure roots may gather evidence for predictions [13]. Nothing other is called for.

With that being said, minimization of prediction error is most likely accomplished through a combination of both perceptual and active inference. Plants perceive and act, and to alternate appropriately between these two modes calls for both the assessment and contextual integration of a number of parameters, and for the capacity to learn. Fortunately, we now know that plants can do both [4,40]. In fact, we have considered, for simplicity's sake, the perception of a single modality (the perception of salt), but salt-avoidance behaviour cannot be assimilated into a response to environmental stimuli on a one-to-one basis. The halotropic behaviour observed in plant roots is not a hard-wired salt-avoidance response [41]. One should bear in mind that plants' adaptive behaviour constitutes an integrated response to many different exogenous and endogenous signalling factors, both biotic and abiotic [8]. For instance, root halotropism permits seedlings to bypass patches of salt while weakening or inhibiting the response to gravity and light [41–43]. As aforementioned, plant behaviour is soft-wired and is reversible. It is the implicit multimodal integration that accounts for the adaptive responses observed. On the other hand, we must bear in mind that plants cannot really afford metabolically costly mismatches. Developmental modifications and phenotypic plasticity take time; with changes taking place over time scales that range from minutes or hours to days and seasons. They cannot thus afford poor decision-making. Plants must adjust with respect to future conditions if their behaviour is to remain adaptive [7,11].

Finally, optimal anticipation requires some form of learning, however basic, that allows the plant to sculpt its internal model. Although at first sight this may look like one of the most difficult aspects for plants to deal with, recent findings suggest that plants may be capable of learning [44,45]. It is thus possible that forms of plant learning may play the role needed, and that overall integrated signal assessment (e.g. updating plants' inner models in order to generate predictions in contexts in which salinity interacts with photo- and gravitropic responses) can be approached *cognitively*.

The basic toolkit of plant neurobiology and predictive processing as herewith sketched may allow us to rethink more carefully what predictive processing actually implies. Note that once we consider plants—*non-neuronal* organisms—a more radical picture begins to emerge. On the other hand, according to a more conservative approach to PP [46], action is not considered to make such a big difference with respect to the basic inferential Helmholtzian framework (sketched in §2). Were plants to make good predictions, and the empirical evidence appears to suggest so, it must be due, someone may wish to argue, to the fact that plants have evolved an ‘internal mirror of nature’ [47] that serves their purposes.

In what follows, we discuss how radical PPP could be—by taking issue with the idea of plants mirroring nature and therefore departing from more conservative PP scenarios. In particular, we shall elaborate on the following two aspects to pave the way for a really radical PPP approach: adaptive behaviour that while non-neuronal still provides the substrate for bidirectionality and functional asymmetry (§4); and plant frugality and embodiment (§5). General conclusions and discussion about the perception of affordances by plants—as directions for future research—will follow (§6).

## 4. Non-neural message passing and belief propagation

Plants, like animals, live surrounded by uncertainty. But how can plants possibly accomplish prediction error minimization? In brief, bidirectional, functionally asymmetric flows of processing are needed to generate predictions and encode prediction errors [48]. In predictive coding architectures, this usually entails a downward flow of predictions to sensory organs or receptors that is reciprocated by an upward flow of prediction errors that update or adjust physically encoding expectations (that generate the descending predictions: see §6.3). In other words, if a sensory mismatch occurs, an error signal propagates upwards, to update internal states of the plant or creature to account for the discrepancy detected. So, it seems that all these processing flows are going to require some serious machinery. In mammals, the neural substrate for this requisite message passing is pretty handy, with fast neuronal message passing among the levels of brain (i.e. cortical) hierarchies by axonal processes. But whether this neuronal machinery sufficient or not is not the question. Is it necessary?

Fortunately, many organisms may well have the type of machinery required for predictive coding. Neocortices can certainly do a lot with their hierarchical structuring, bringing abstraction beyond that dreamt of for other species. This however does not mean that something mammalian or neuronal is needed. What is not negotiable is the functional (predictive) processing *itself*, not the substrate details of implementation. As it turns out, the internal system of plants is, in some important respects, similar to the animal nervous system [49], and although the evidence as to how the free-energy principle is actually implemented in plants is still forthcoming [13], we may wonder what sort of non-neural substrate could underlie PPP.

Plants appear to have all the necessary machinery to implement the free-energy principle. Plant neurobiology is beginning to unveil the type of functionally equivalent machinery in terms of analogues to layered interconnected patterns of neural firing. Although plants are not equipped with neurons, Darwin drew a felicitous analogy between the root tip and the brain of lower animals that resists the passing of time:

It is hardly an exaggeration to say that the tip of the radicle [root] thus endowed, and having the power of directing the movements of the adjoining parts, acts like the brain of one of the lower animals; the brain being seated within the anterior end of the body, receiving impressions from the sense-organs, and directing the several movements [50].

In fact, phytoneurological structures that play a role analogous to the nervous system of animals have long been identified. As J.C. Bose documented in *The Nervous Mechanism of Plants* [51], some decades after Darwin's major work in botany [50,52,53], there is transmission of excitation between the petiole and the pulvinus of *Mimosa pudica* that results in loss of turgor, and subsequent leaf folding. But electric communication is spread throughout the plant kingdom, beyond the rapid responses of *Mimosa pudica*, Venus flytrap, and the like. Plants, like animals, fire spikes of voltage. Plant firing may be triggered by a variety of signals, such as herbivory, variations in illumination and temperature, mechanical stimulation or salt stress, among many others. These can result, for example, in changes in photosynthesis, respiration, or in gene expression. Such firing—action potentials (APs) and variation potentials (VPs), also known as slow wave potentials (SWPs) [1,54]—plays a central role in integrating the plant internal states via the propagation of electric signals. Both APs and VPs (SWPs) share with animal APs their threefold electrophysiological profile of depolarization–repolarization–hyperpolarization [55], with electric signals propagating over short distances through plasmodesmata (the plant equivalent to intercellular gap junctions in animal tissues), and travelling over long distances—at least, in the case of APs—along the vascular system (phloem) in both directions.^3^

Cellular electrical excitability for the purpose of the transmission of information relies upon the capacity of plant cells to conduct such signals from receptor to effector sites, despite the lack of a central nervous system proper [49]. Electrical events can propagate in the membranes of neural and non-neural cells alike. In the absence of axons or other projections for the purpose of conduction, electrical events propagate in the membranes of plant cells along the vascular system. Neural-like signalling through the vascular system of plants—a system that stretches throughout the plant body from root to shoot in the form of vascular bundles of phloem, xylem and cambium [59]—underlies in part their phenotypically plastic responses. Signal integration is implemented, at the electrical level, via long-distance electrical signalling. Certain neurotransmitter-like chemicals, and the transport of auxin as well as other phytohormones [2], underpin the capacity to integrate signals at the chemical and molecular levels.^4^ So, we can see that despite the fact that plants lack neurons, they can respond in a fast and coordinated manner to environmental contingencies.

With that being said, if the PPP hypothesis is correct, bidirectional and functionally asymmetric message passing ought to be observed (we take the reciprocal and asymmetric message passing of bottom-up prediction errors and top-down predictions, as exemplified by mammalian neocortical structures, to be non-negotiable [12]). Importantly, electric communication can take place over long distances through vascular bundles located within the transport system of vascular plants, both top-down and bottom-up. On the other hand, horizontal lateral signalling over short distances takes places through plasmodesmata [56]. Signals passing through local plasmodesmata networks can thus reach long-distance vascular pathways. In this way, plants seem to have the type of informational pathways required to implement PPP.

As in the animal neurobiology literature, plant neurobiology entertains the idea of a hierarchical arrangement; in terms of the distinction between bottom-up, forward and top-down, backward connections. The requirement of functional asymmetry, however, does not entail that the different *types of* non-neural cells are needed. Vascular cells may ‘simply’ exhibit distinct dynamics depending on whether they mediate predictions or encode error. But the way to propagate top-down and bottom-up signals can vary a lot. Plants may implement PPP principles either by means of other substrates/cell subpopulations for the same prediction and error flows, or even exploiting different types of flows of predictions and errors altogether.

On the other hand, the distinction between forward and backward connections may be based upon the specificity of vascular tissues that connect the overall vascular network of plants, and of intrinsic connections found within each vascular tissue. One possibility worth exploring is provided by phloem vascular and apical cells, respectively, exhibiting different functionalities. Distinct vascular cell populations may encode expected states of the world and prediction error, respectively. It is possible that vascular cells in deep phloem layers serve to encode the former (i.e. mediate predictions), and apical cells encode the latter (i.e. convey prediction errors). We elaborate further in §6.

It should also be pointed out, as noted in [24]: ‘even non-neuronal cells possess many of the same ion channel- and electrical synapse-based mechanisms as do neurons and use them for pattern formation and repair’ [60–62]. As to the way in which cellular dynamics encode particular states, it is worth noting that although the role of acetylcholine, glutamate, dopamine, serotonin, and other neurotransmitters found in plants still needs to be clarified [49,57], the possibility that in analogy to the animal neuron, acetylcholine or dopamine, for example, underlie modulation in neural-like tissue cannot be discarded. The functional analogues of signalling molecules, across the plant and animal kingdoms, have been disclosed recently by the study of neurotransmission in plants. γ-Aminobutyric acid, for instance, has been primarily studied in its metabolic role, although see [63]. From that perspective, cellular signalling looks pretty much the same in animal neural and plant networks. In fact, the way cellular signalling is optimized predates the evolution of central nervous systems in mammals and the vascular system of plants. Early in evolution, cellular signalling in somatic networks found a way to coordinate physiological needs, so that their behaviour could remain adaptive [64].

The simulations reported in [24] are particularly prescient for the current argument. These simulations used an ensemble of free energy minimizing ‘cells’ that employed chemotaxis to self-organize and emulate morphogenesis. Crucially, the chemotactic signals were generated by the cells themselves, providing a form of self-assembly that was mediated by predictive processing; namely, non-neuronal inference about where each cell was located in relation to others. In this example, predictions and prediction errors were encoded by intracellular macromolecules and extracellular electrochemical gradients. One way or another, our working hypothesis is that error correction is implemented by biophysical processes within vascular structures [8] that (spatially) organize an exchange with the world that complies with active inference.

Overall, different organisms may have evolved alternative strategies to appropriately combine top-down, bottom-up, and lateral flows. Non-neural patterns of connectivity may thus allow plants to predict sensory states. The capacity to anticipate may be implemented for instance in temporal patterns of synchronous oscillatory firing of specific populations of plant cells [13]. In short, if animal synaptic activity can bring about active inference, the same in principle applies to the electrosensitive cells of plants.

## 5. Embodiment and frugality

According to the foregoing, plant cognition can be grounded in the absence of a neural-based generative model. But by the same token, strictly speaking, plant cognition is not grounded either (exclusively) in a non-neural-based functional analogue. Cognition extends throughout the substrate of plants, their body and the environment. In this way—focusing upon the plant body—facts about morphology and the material composition of plants are crucial. Consider the ‘Yokoi hand’ [65]. This prosthetic hand was designed with flexible and soft gripping materials that, courtesy of a particular morphology, could grip a variety of objects. Importantly, both morphology and materials deliver the goods—reducing the amount of predictive control that would be otherwise required. A morphological design embodies the coming together of the finger tips as the hand itself closes. On the other hand, the type of material—that the finger tips are made of—fits hand in glove with the type of objects to be gripped.

If a robotic hand can exhibit a degree of adaptation to different shapes and contingencies through its material constituency and morphology, the same can be said of plants. Plants, we may say, compute with their bodies in the service of adaptive flexible behaviour. Consider a vine climbing up a host tree for photosynthetic purposes. Vines can attach to supports in many different ways; insofar as attachment mechanisms and stem structure and function are concerned. Their capacity to cling to host trees resembles pretty much the type of extended solutions found by morphological roboticists. In fact, depending on the type of attachment, vines are able to climb some supports and not others. Thus, tendril and twining climbers have more efficient climbing mechanisms than hook-climbers [66]. The stems of the former are more flexible; those of hook-climbers, more rigid. As a result of their very phenotypic composition, some climbers can cling to supports of different diameters. Some redvines make use of jelly-like fibres allowing the tendrils to squeeze supports. Importantly, depending on the direction of coiling, the tendrils have their gelatinous fibres distributed in different ways (e.g. those that coil always in the same direction have all the fibres on the concave side [66]). Other anchoring strategies rely on glandular secretions, widening the range of supports potentially climbable [52].

The mechanoreceptor tactile bleps of tendrils of *Bryonia dioica* Jacq [67] illustrate nicely one way in which sensory and effector organs act synergistically in this sort of embodied (active) inference. The high density of plasmodesmatal connections in tendril epidermal cells underwrite the electrochemical coordination required for appropriate coiling. But tendrils do not just respond to pressure. As originally observed by Pfeffer and Haberlandt [67], sliding movements on a rough surface are needed. In short, materials and their properties are put to service in the perception of gradients.

The Yokoi hand–vine plant metaphor takes us to the idea of frugality. Plants are good at deploying *the least complex* model, form or generative model that will serve their needs. Recall from above that the overarching imperative prescribed by the free-energy principle is a resolution of uncertainty. If we unpack this in anthropomorphic terms, it simply means that actions (at least good actions) will resolve uncertainty. This is synonymous with selectively gathering evidence for one's own existence and choosing behaviours that have the greatest epistemic value [28,46]. This brings with it the important notion of *epistemic affordance*. In other words, sentient plants and creatures behave in a way that resolves uncertainty about the environment through some form of epistemic foraging. This sort of affordance goes beyond minimizing surprise (e.g. by avoiding high salt concentrations); in the sense that the opportunity to resolve uncertainty now becomes attractive (e.g. deliberately sampling some unexplored part of the environment to see whether it has a high or low salt concentration). One can see immediately that this is just a way of describing exploration and the intrinsic (epistemic) value of certain behaviours that affords the opportunity to reduce uncertainty (i.e. exploit novelty).

## 6. General discussion and directions for future research

### 6.1. The functional anatomy of plants

From the free-energy principle, it follows that plants represent the causes of sensory stimulation in terms of their internal states and morphology. Anatomical and physiological constraints underlie the capacity of plants to do so. By analogy with Friston [68], an optimization scheme under a free-energy formulation allows us to account for a number of empirical aspects at the level of both plant anatomy and physiology, as summarized in table 1. These aspects range from the hierarchical arrangement of vascular tissues and the functional asymmetries between forward and backward connections to minimal cognition as discussed in plant neurobiology [69]. In table 1 we consider three levels of explanation and predictions: (i) at the level of plant anatomy and connectivity, the hierarchical deployment of plant vascular tissues and connections; (ii) at the level of plant electrophysiology, phenomena like repetition suppression; and (iii) at the psychophysiological level, the working hypothesis of plant neurobiology is that it can explain cognitive level phenomena, such as plant priming [7] and learning [70] (table 1 for details on domains and predictions).

**Table 1.** Structural and functional aspects of the plant vascular system that may be explained under a free-energy (predictive processing) formulation.

domain	prediction
*anatomy and connectivity*: explains the hierarchical deployment of plant vascular bundles, architectures with forward and backward (bidirectional) connections [1,54,55,57,58]	—hierarchical vascular organization —distinct vascular cell populations, encoding expectations and prediction error. Crucially, these distinct populations should be reciprocally connected because, algorithmically, every biophysical encoding of an expectation passes messages or signals to an associated prediction error population and vice versa —forward connections convey prediction errors from mechanoreceptors and chemoreceptors (e.g. on apical cells) and backward connections mediate predictions (e.g. from deep vascular cell bundles) —functional asymmetries in forwards (linear) and backwards (nonlinear) connections are mandated by nonlinearities in the generative model encoded by top-down backward connections conveying predictions —vascular cells elaborating predictions (e.g. deep vascular cell bundles) could show distinct (low-pass) dynamics, relative to those encoding error (e.g. cells in root and shoot apices). This follows from the fact that expectations accumulate evidence from prediction errors; thereby suppressing fast (high-frequency) fluctuations in prediction errors —recurrent dynamics are intrinsically stable because they suppress prediction error. In other words, if cells encoding errors excite cells encoding expectations, expectations should inhibit errors—or vice versa
*electrophysiology*: explains the prevalence of action potentials (APs) and variation potentials (VPs), also known as slow wave potentials (SWPs), in plant electrophysiology [54–58]	—sensory responses are greater for surprising, unpredictable or incoherent stimuli (e.g. sudden changes in salt concentration or mechanical stimulation) —the attenuation of responses encoding prediction error, with perceptual learning. In other words, we would predict that regular fluctuating mechanical, photic or chemical stimulation will entrain plant electrophysiology—and that these induced responses should decay with repetition—and re-emerge with novel stimuli—or, importantly, an omission
*psychophysiology*: accounts for the behavioural correlates (e.g. growth and phenotypic changes) of physiological phenomena [1]	—predictive processing furnishes a framework in which to model and understand priming and learning phenomena in plants of the sort that underlies omission related responses (see above) and experience dependent plasticity in the way top-down predictions are formed

Consider first the hierarchical vascular organization of plants. Plants are characteristically modular, with a highly decentralized architecture. This reflects the evolutionary needs of sessile organisms, for which a highly centralized system would not be adaptive. Yet, a hierarchical organization is needed if predictions and prediction errors are to flow up and down the plant body. In fact, the modular decentralized architecture of plants is compatible with a considerable degree of hierarchical organization. For example, vascular cells are arranged in layers that converge at both ends, at the root and shoot apices [71]. At a different level of description, individual modules in shoots are connected by nodes, and the same goes for every anatomical subunit of the entire plant body. This enables modules to connect to each other for the purpose of signalling [72]. Overall, vertical xylem and phloem strands, which are highly cross-linked by horizontal and tangential anastomosis [59], form a ‘channel and net’ or reticulated structure that runs throughout the plant [1,55,72,73].

With regard to the electrophysiological and psychophysiological domains (table 1), we may consider repetition suppression and learning [13], respectively. Repetition suppression, a phenomenon in which responses are attenuated as a result of the repeated presentation of a particular stimulus, may well take place in plants, with top-down expectations underlying suppression. With respect to learning, habituation [44] and associative learning [45], these may well play a functionally equivalent role in plants, in the service of generating predictions that are informed by a plant's experience.

### 6.2. The theoretical biology of plants

The two principal themes of functional asymmetry (between descending predictive and ascending prediction error signals) and the frugal nature of a plant's functional architecture speak to the two fundaments of free-energy minimization outlined in this article. In particular, the frugal, complexity minimizing structure of plants—well suited to their world—follows naturally from the minimization of free energy. This follows from the mathematical equivalence between free energy and (Bayesian) evidence for a generative model [16]. In other words, if we associate the structural and dynamical form of a plant with a model of its eco-niche, then any process that minimizes free energy will necessarily maximize model evidence. Because the plant *is the model*—it is basically self-evidencing [46].

This can be seen at a practical level in everyday statistical analysis where it is known as Bayesian model selection. The same arguments have been applied in the context of evolution—such that selective pressure is simply the process of free-energy minimization playing out at an evolutionary time scale [74–76]. In short, natural selection is nature's way of performing Bayesian model selection. A key insight here is that *evidence is the difference between accuracy and model complexity*. In other words, a good model with high evidence will provide an accurate explanation for sensory exchange with the environment, while minimizing its complexity.

Complexity can be thought of as the degrees of freedom used by the plant to anticipate and predict its sensory exchange. This leads naturally to a principle of minimum redundancy (well established in the neurosciences [77]), whereby a good plant will retain just those sparse, frugal structures that are necessary to anticipate the world. This can be evident in the phenotypic form (as unpacked earlier by analogy with robotic gloves) or in terms of conditional dependencies and ‘action at a distance’ mediated in plants by channels and electrochemical waves (very much like axonal connections and electrochemical synaptic transmission in the brain). In short, under the free-energy principle—and the active inference that this entails—one would anticipate that plants would come to distil the essential causal structure in their environment in terms of their physical form and biophysical function. It is this form and functional architecture that constitutes the generative model and underwrites their existence.

### 6.3. Predictive coding in plants

The dynamics within this generative model (e.g. plant) correspond to signalling of the sort associated with belief propagation in the brain. This implicit (Bayesian) message passing is simply a description of dynamics that maximize Bayesian model evidence (or minimize free energy). This can manifest in many forms. A popular example is predictive coding in the brain [17,18]. The crucial aspect of these dynamics (variously known as variational message passing, belief propagation, predictive coding etc.) is an asymmetry in the messages that are passed on the internal states generating predictions. Interestingly, one can always formulate a free energy minimizing scheme in terms of prediction errors, if the generative model calls upon probability distributions within the exponential family [78]. This is just a technical way of saying that it is more than likely that any plant or creature can be described (at least mathematically) as passing predictions and prediction errors around its body in the service of minimizing prediction error.

Finally, it is worth noting that the very idea of plant perception seems to turn upside down our very intuitions as to what perception and action entail. In this context, one can consider directions for future research in the context of plant action and the perception of affordances. If plants are sentient and behaving organisms, then plant perception and action are driven primarily by anticipatory routines triggered in the plant's body. At this point we can see how radical PPP can actually be. Plants are surprised by the unexpected, and their capacity to navigate uncertain terrains may therefore depend on a cascade of top-down predictions, constantly predicting incoming signals. As in the case of pea roots that can predict the future [7], anticipatory behaviour is crucial for adaptive, surprise eluding, success.

Vines provide the sharpest contrast with conservative PP scenarios. Grasping, say, a phone, according to Hohwy [46] can only be explained via the exploitation of an internal representation of the phone itself. But once PP is fully developed with fast and frugal, morphological, and non-neurally decentralized resources, we can see that detailed internal representations may not be necessary: a climbing plant does not grasp a support because it has an internal representation of it, but rather because of the soft-assembling of the plant body–environment in the context of the approaching manoeuvres. In other words, the tendrils of the plant do not call upon representations of the affordance to climb: they are, in and of themselves, the generative model which realizes that affordance.

The very exploitation of ecological information within a PP framework permits us to envisage really radical predictive processing solutions like those be leveraged by plants. That plants can perceive ecological information is not the big news. Turvey *et al.* [79] considered how *Monstera gigantea*, a climbing vine, could perceive a form of climb-ability, as this plant would grow skototropically towards darkness [5]. Thus, vines can perceive possibilities for action, but to fully understand this ‘perception’, we need to bear in mind that the vine and its support are functionally coupled by the predictions of ‘experienced’ plants. Vines control their approaching manoeuvre to the support by anticipating sensory states, and realizing the states through embodied action.

Plants are a good example of radical predictive processing because they are not in the business of enriching a neurocentric model of their surroundings (this is effectively outsourced to natural selection); rather, they are in the business of appropriately coupling with it. A movement of circumnutation, for instance, is triggered, maintained and modified endogenously, or at least that is our working hypothesis. Taking into account that many growth-related movements are irreversible, exploration must be accomplished efficiently. Control is thus needed, if the metabolic and computational cost of irreversible but idle movements is to be minimized.

These considerations suggest an understanding of plant cognition radically different—not only with respect to mainstream cognitive science, but also to conservative approaches to PP. When we say that plants are proactively engaged with their surroundings we mean literally that they foresee possibilities of interaction with their local environment ahead of time. But it is important to emphasize that this needs to be so. A plant that only represents sensory inputs as they flow past would be *dead* meat. This is so precisely because they move slowly and cannot afford to react reflexively in response to the present. Plants, despite the slowness of their responses, can exploit distal nutrient-related or reproduction-related causes in their eco-niche. In short, they need to deal with contingencies ahead of time—because they cannot afford to behave otherwise.

## Endnotes

1 In [13] the ‘predictive processing’ hypothesis (see §2) was contrasted against ‘feature detection’ in the canonical domain of mammalian visual cognition, transferring the rationale involved to the plant literature.

2 This is not only in the case of plants, but applies more generally. Just think for a moment of halophilic archaebacteria (‘salt bacteria’). They thrive in salt lakes or evaporation ponds under extremely harsh conditions of salinity. Less dramatic scenarios are those of organisms such as marine macroalgae and phytoplankton species [36].

3 VPs are generated at the plasma membrane of parenchyma cells, and it is possible that they also can pass through the plasmodesmal network and reach the phloem pathway [56]. For reviews of plant electrophysiology see [54,55,57,58].

4 We focus on electric signalling for the sake of drawing the neural/non-neural parallel more vividly. Having noted that plants have no neurons, the reader may wish to use the terms ‘phytoneurology’, and ‘phytoneurones’ to refer to excitable phloem cells and electric conduction through them [59]. For a scientific justification of the ‘plant neurobiology’ terminology see [4].
